# Long-term Outcome following Percutaneous Intervention of Intra-stent Coronary Occlusion and Evaluating the Different Treatment Modalities^☆^

**DOI:** 10.1016/j.ijcha.2021.100803

**Published:** 2021-05-31

**Authors:** Sandeep Basavarajaiah, Satoru Mitomo, Sunao Nakamura, Vinoda Sharma, Ishaq Mohammed, Yusuke Watanabe, Toru Ouchi, Gurbir Bhatia, Jerome Ment, Sampath Athukorala, Michael Pitt, George Pulikal, Bethan Freestone, Hannah Rides, Nitin Kumar, Richard Watkin, Kaeng Lee

**Affiliations:** a Heartlands Hospital, University Hospitals Birmingham, United Kingdom; b New Tokyo Hospital, Chiba, Japan; c Birmingham City Hospital, United Kingdom

**Keywords:** In-stent restenosis, Chronic total occlusion, Drug eluting stent, Drug coated balloon, CKD, Chronic kidney disease, ISR, In-stent restenosis, CTO, Chronic total occlusion, TVMI, Target vessel myocardial infarction, TLR, Target lesion revascularisation, TVR, Target vessel revascularisation, MACE, Major adverse cardiac events, LVSD, Left ventricular dysfunction, POBA, Plain old balloon angioplasty, DCB, Drug coated balloon, DES, Drug eluting stent

## Abstract

**Background:**

Angioplasty for ISR remains a challenge with relatively high rates of recurrence. Although there is a plethora of data on ISR, there is relatively less data on intra-stent-CTO. In this study, we explore the long-term clinical outcomes following angioplasty to intra-stent CTO and study the differences in clinical outcomes between three treatment-arms: POBA vs. DES vs. DCB.

**Methods and results:**

We evaluated all patients who underwent PCI to intra-stent CTO between 2011 and 2017. The endpoints used were: cardiac-death, TVMI, TLR, TVR, and MACE.

During the study period, 403-patients with a mean age of 69.2 years had successful PCI to intra-stent CTO. 50% were diabetic, 38% had CKD and 32% had left ventricular dysfunction. 93% of cases were stable angina. 22% (n = 88) received only POBA, 28% (n = 113) received DCB and 50% (n = 202) received DES. During the median follow-up of 48-months, cardiac-death occurred in 5.8% (n = 23), TVMI in 4% (n = 16), TLR in 45.6% (n = 182), TVR in 48.7% (n = 194) and MACE of 46%. There were no differences in the hard endpoints between the 3treatment arms. However, the TLR and overall MACE were better in DCB and DES-groups as compared to POBA (TLR: 33%vs.42%vs.49%; p = 0.06); MACE (34% vs. 45% vs. 52%; p = 0.05).

**Conclusion:**

This is the first study that has focussed on the outcomes following angioplasty to intra-stent CTOs with a very long-term follow-up. The hard endpoints were low, although the TLR rates were high. In regards to treatment strategy, the DCB and DES provide relatively better outcomes than POBA.

## Highlights

• Limited data on intra-stent CTO.
• We explored the long-term outcomes in patients undergoing PCI to intra-stent CTO.
• Acceptable rates of hard endpoints (cardiac death; 5.8%, TVMI; 4%).
• High rates of TLR (45.6%).
• PCI should only be undertaken if symptomatic or if there is inducible ischaemia.
• If PCI is undertaken, DCB and DES should be considered over POBA.

## 1 Introduction

Percutaneous coronary intervention (PCI) has come a long way since its introduction with consistent improvements in device technology and pharmacotherapy that has resulted in considerably low event rates [1], [2]. However, despite these improvements, in-stent restenosis (ISR) remains a common long-term problem of angioplasty; in-fact, the frequency of ISR escalates with both patient and lesion complexity [3], [4], [5]. The treatment of ISR is very challenging with high rates of recurrence irrespective of the modality of treatment [6], [7], [8]. The current recommendations from the European Society of Cardiology is to choose either drug coated balloon (DCB) or second-generation drug eluting stents (DES) [9]. Plain old balloon angioplasty (POBA) is still in practiced in certain centres especially in the United States, where the use of DCB in coronary intervention is not licensed for routine clinical use. Most of the available data to establish such guidelines have been extrapolated from studies evaluating different treatment modalities for ISR [8], [10], [11], [12]. However, occlusive restenosis (type IV on Mehran’s classification) [13] which has TIMI 0 flow or otherwise referred to as intra-stent CTO is either excluded or poorly represented in such studies. Although there are published studies on intra-stent CTO, there is a lack of data on long-term clinical outcomes [14], [15], [16]. In addition, no studies have compared the different treatment modalities (POBA vs. DES vs. DCB) in this complex group of ISR. In this study, we have retrospectively evaluated all patients who underwent PCI to intra-stent CTO at 2 high-volume centres (in Japan and United Kingdom) and report its very long-term follow-up.

## 2 Methods

We retrospectively evaluated all patients who underwent successful PCI for intra-stent CTO between January 2011 and December 2017 at 2high-volume centres (Heartlands Hospital, Birmingham and New Tokyo Hospital, Tokyo). The CTO was defined as a completely occluded artery within the previously stented segment with TIMI 0 flow and the duration of the occlusion of >3months. We only included patients who presented with occlusive restenosis for the first time and patients who previously had their intra-stent CTO treated and re-presenting as new occlusion were excluded. Patients were divided into 3 groups based on the treatment modality they received (POBA, DES and DCB) and were compared for baseline characteristics and clinical outcomes. Since the follow-up between the 3 treatment arms were not uniform, we have reported the clinical outcomes censored at the time of first event or at 24months, whichever occurred first. Events occurring after 24months were not counted and anyone with less than 24months follow-up were excluded from the analysis. If patient received bailout stenting after DCB, they were included in the stented group for the analysis. The study has received appropriate ethical approval from the respective local institutions to access patient data.

## 3 Procedure

All patients were pre-treated with aspirin (300 mg loading when needed) and clopidogrel (loaded with 300mg) or new P2Y12 inhibitors (ticagrelor or prasugrel) in acute coronary syndromes. Unfractionated heparin was administered at a dose of 70–100 units/kg. Procedural techniques for CTO-PCI (antegrade or retrograde) including use of adjuvant kits were left to the discretion of the operator. After successful wire passage, lesions were pre-dilated with any of semi-compliant, non-compliant, scoring and/or cutting balloons (alone or in combination), and rotational atherectomy was used if required. Subsequent treatment with POBA or DCB or second-generation DES was left to the operator. If DCB was considered, it was inflated at nominal pressure for 60s to aid drug delivery. Patients treated with only DCB received dual anti-platelet therapy for a minimum of 1month post-procedure (unless if it was in the setting of acute coronary syndrome in which case it was extended to at least 12 months). If patients received DES, dual anti-platelet therapy was prescribed for a minimum period of 6–12 months as per the current guidelines. All patients were advised to continue lifelong aspirin (75 mg) or clopidogrel (75 mg).

## 4 Follow-up

Follow-up was achieved through clinic visits, telephone calls, and written correspondence from general practitioners and records from hospital admissions. Events were adjudicated by the interventional cardiologists and fellows who were not part of the index procedure. The measured endpoints during this follow-up were: death from any cause, cardiac death, target vessel myocardial infarction (TVMI), target lesion revascularisation (TLR), target vessel revascularisation (TVR), stent thrombosis (definite and probable) and major adverse cardiac events (MACE). Death was considered cardiac in origin unless obvious non-cardiac causes could be identified. TVMI was defined as an elevation of troponin above the upper range limit in combination with at least one of the following: symptoms of ischemia; ECG changes indicative of new ischemia; or the development of pathological Q waves on ECG. When out-of-hospital MI was diagnosed clinically, it was coded as target vessel MI unless coronary angiography demonstrated an acute occlusion within a vessel that was not treated during the index procedure. When angiography was not performed, or there was doubt on angiography as to the culprit vessel, the event was coded as an TVMI. TLR was defined as any revascularisation of the target lesion (within the previously-treated segments) driven by: a positive functional ischemia study, ischemic symptoms, and a diameter stenosis ≥70%. TVR was defined as PCI or surgical revascularization of the target lesion or any segment of the epicardial coronary artery containing the target lesion. The MACE rate was defined as combination of cardiac death, target vessel MI and TLR [17]. Stent thrombosis was categorised according to the definitions proposed by the Academic Research Consortium [18].

## 5 Statistics

Continuous variables were presented as mean (±SD) and compared by single factor analysis of variance (ANOVA). Categorical variables were presented as percentage and compared by chi-squared test. MedCalc^@^ was used for statistical analysis. Missing values were replaced by mean of nearest neighbours. Values with >5% of missing values were excluded from the analysis. Time to first events (cardiac death, TLR and MACE) were plotted on Kaplan Meier curves and compared by log rank p value.

## 6 Results

During the study period 452lesions (intra-stent CTO) were attempted of which 403 were successful (89%). In the 403 successful cases; the mean age was 69.3 years and 336 (83%) were male. The complete demographic characteristics are provided in Table 1, 51% (n = 204) were diabetic, 38% (n = 152) patients had CKD, which was defined as estimated glomerular filtration rate (eGFR) of <60 mL/min. Most of the procedures were undertaken in the setting of stable angina (n = 376; 93%) and 7% were in the setting of ACS. All patients with ACS were unstable or crescendo angina and none were ST-segment or non-ST segment elevation myocardial infarction to suggest acute occlusion. Left ventricular dysfunction (defined as ejection fraction <50%) was seen in 129 (32%) of patients.

**Fig. 1:**
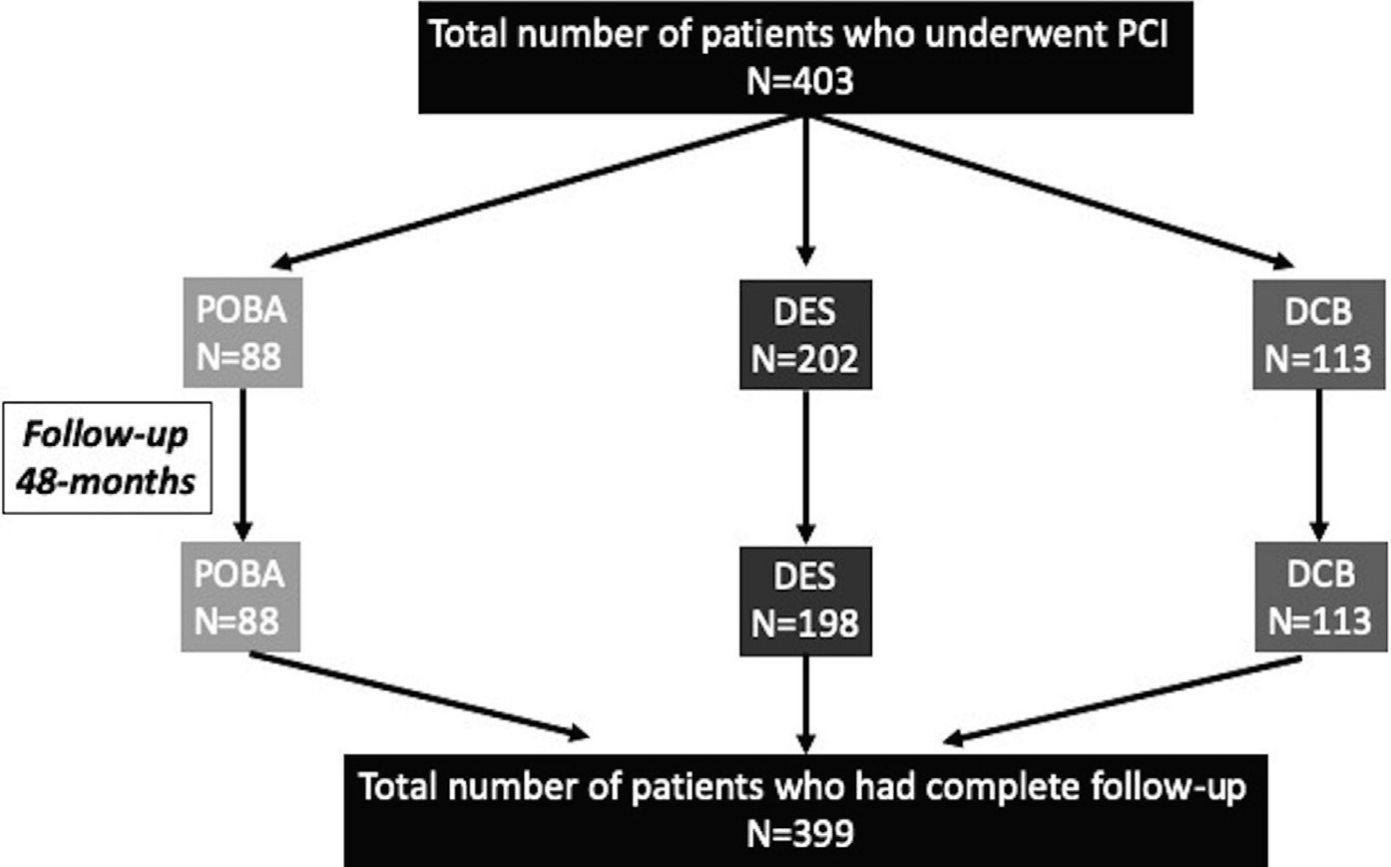
Flow-chart demonstrating the total number of patients included, treatment modalities received and total number for analysis after follow-up.

Procedural characteristics are provided in Table 1. 83-patients (21%) were ISR in a previously placed BMS and 321 patients (79%) were ISR in previously placed DES. Of the 82patients with BMS-ISR; 22% (n = 18) had POBA, 12% (n = 10) had DCB and 66% (n = 54) received DES. Of the 321patients with DES-ISR; 22% (n = 70) had POBA only, 32% (n = 104) had DCB and 46% (n = 147) received DES. An antegrade approach was used in the vast majority of cases (n = 397; 98.5%) with only 6 cases needing retrograde technique to complete the case. Non-compliant balloon pre-dilation was used in 93% (n = 376) of cases, with scoring and cutting balloon in 8% (n = 33) and 3% (n = 13), respectively. Rotational atherectomy was used in 19cases (5%). After successful pre-dilatation, 88 cases (22%) received no further treatment and were classified as POBA. 113cases (28%) were treated with drug coated (Paclitaxel) balloons, and 202cases (50%) with second generation DES (Fig. 1). Intravascular imaging to elucidate the mechanism of occlusion was undertaken in 363 cases (90%).

**Table 1:** Demographic and Procedural characteristics.

Demographics	N = 403
Age (Mean ± SD)	69.2 ± 9.6
Male	333 (83.5%)
History of smoking	153 (38%)
Hypertension	319 (79.9%)
Diabetes Mellitus	201 (50.4%)
Insulin dependent diabetes mellitus	48 (12%)
Chronic kidney disease	151 (37.8%)
Stable angina	372 (93.2%)
ACS	31 (7.7%)
Previous CABG	24 (6%)
Left ventricular systolic dysfunction	129 (32.3%)
Previous stent (BMS) • *Treated with POBA* • *Treated with DCB* • *Treated with DES*	83 (21%) 18 (22%) 10 (12%) 54 (66%)
Previous stent (DES) • *Treated with POBA* • *Treated with DCB* • *Treated with DES*	321 (79%) 70 (22%) 104 (32%) 147 (46%)
Antegrade CTO PCI technique	397 (98.5%)
Retrograde CTO PCI technique	6 (1.5%)
Intravascular imaging	363 (90%)
Predilatation	398 (99.7%)
Non-compliant balloon	372 (93.2%)
Scoring balloon	33 (8.3%)
Cutting balloon	13 (3.3%)
Rotational atherectomy	19 (4.8%)
POBA only	88 (22%)
DCB	113 (28%)
DCB length (Mean ± SD)	48.12 ± 25.7 mm
DES	202 (50%)
DES length (Mean ± SD)	55.58 ± 28.8 mm

Of the 403cases, only 399 were included for analysis of clinical outcomes as follow-up was not available for 4 cases (Fig. 1). Details of clinical outcomes are provided in Table 2. During a median follow-up of 48 months (Interquartile range; 28–67 months), cardiac death was seen in 23cases (5.7%) and target vessel MI in 16cases (4%). Target lesion revascularisation was seen in 182 cases (45%) and TVR in 194 (48%) cases. The overall MACE rate was 46% driven principally by TLR. Stent thrombosis (definite and probable) occurred in 4 cases of the 203 patients treated with DES. Since the follow-up was available in only 198 of the DES cases, the rate of stent thrombosis was 2.5% (n = 5). Angiographic follow-up was available in 289-cases (72%).

**Table 2:** Clinical follow-up.

Clinical follow-up	N = 399
Angiographic follow-up	289 (72%)
Death	70 (17.5%)
Cardiac death	23 (5.8%)
TVMI	16 (4%)
TLR	182 (45.6%)
TVR	194 (48.7%)
MACE	184 (46%)
Stent thrombosis (definite and probable)	5 (2.5%)

**Fig. 2:**
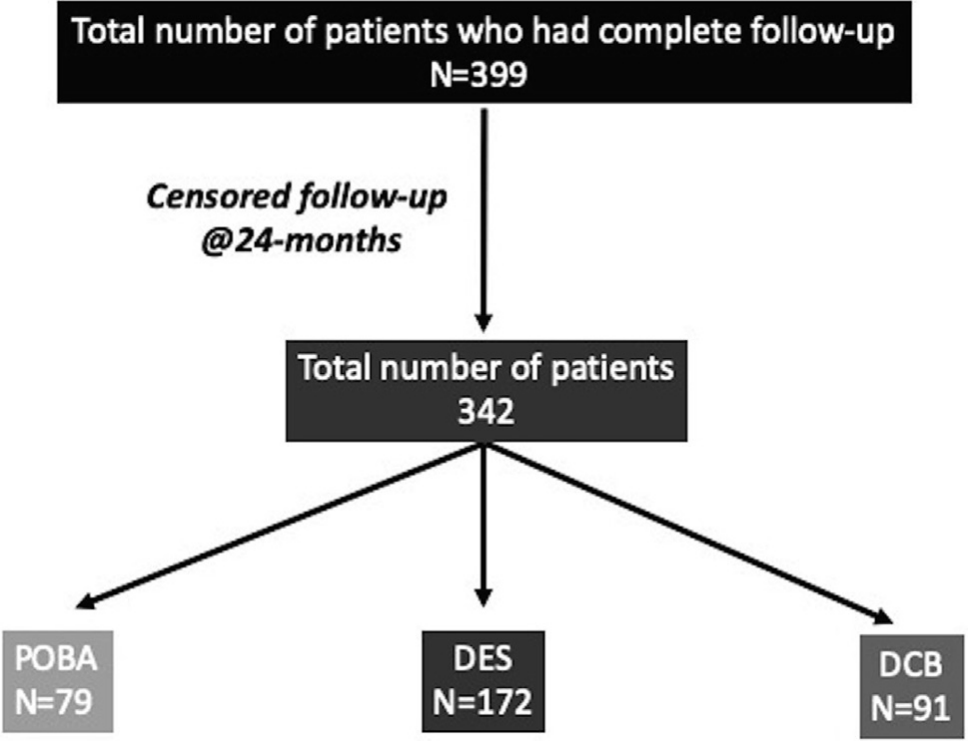
Flow-chart demonstrating the total number of patients included after censoring the clinical events at 24-months.

We have also analysed the differences in the demographic and clinical outcomes between the treatment groups (POBA vs. DCB vs. DES). Since the follow-up were not uniform, we have reported events censored at 24months for all the 3 groups and have excluded those patients who had <24months follow-up from the date index procedure. We had 342patients (79POBA, 172DES and 91DCB) who met the criteria for analysis (Fig. 2). There were no differences in the demographic characteristics between the 3 groups (Table 3). However, there were differences in the procedural characteristics. The use of scoring balloons were significantly higher in the DCB group as compared to POBA and DES. The cutting balloons were used more commonly in the POBA group. There were no differences in the use of non-compliant balloons and rotational atherectomy (Table 3). The DCB diameter were significantly smaller than the DES diameter (DCB diameter 2.84 ± 0.50 mm vs. 2.95 ± 0.50 mm, p = 0.02), but length of stents used were longer than the length of DCB (28.37 ± 8.8 mm vs. 26.94 ± 5.8 mm, p = 0.04).

**Table 3:** Demographics and outcomes with events at 24 months post procedure.

	POBA	DES	DCB	p value
Age (years)	69.8 (±8.0)	69.3 (±9.7)	68.8 (±10.2)	p = 0.81
Female	11 (14%)	27 (16%)	9 (10%)	p = 0.42
HTN	65 (82.3%)	139 (80.8%)	74 (81.3%)	p = 0.96
DM	45 (57.0%)	83 (48.3%)	38 (41.8%)	p = 0.14
CKD	31 (39.2%)	66 (38.4%)	29 (31.9%)	p = 0.51
Stable angina	77 (97.5%)	162 (94.2%)	89 (97.8%)	p = 0.30
LVSD < 50%EF	27 (34.2%)	51 (29.7%)	28 (30.8%)	p = 0.78
Non-compliant Balloon	85 (96.6%)	182 (91.9%)	104 (92.9%)	p = 0.34
Scoring Balloon	4 (4.5%)	7 (3.5%)	22 (19.6%)	p < 0.0001
Cutting Balloon	7 (8.0%)	3 (1.5%)	3 (2.7%)	p = 0.017
Rotablation	3 (3.4%)	7 (3.5%)	9 (8.0%)	p = 0.16
Cardiac death	3 (3.8%)	6 (3.5%)	1 (1.1%)	p = 0.5
TVMI	0 (0%)	3 (1.3%)	0 (0%)	p = 0.2
TLR	39 (49.4%)	73 (42.4%)	30 (33.0%)	p = 0.09
TVR	42 (53.2%)	76 (44.2%)	32 (35.2%)	p = 0.06
MACE	41(51.9%)	77(44.8%)	31(34.1%)	p = 0.05

**Fig. 3:**
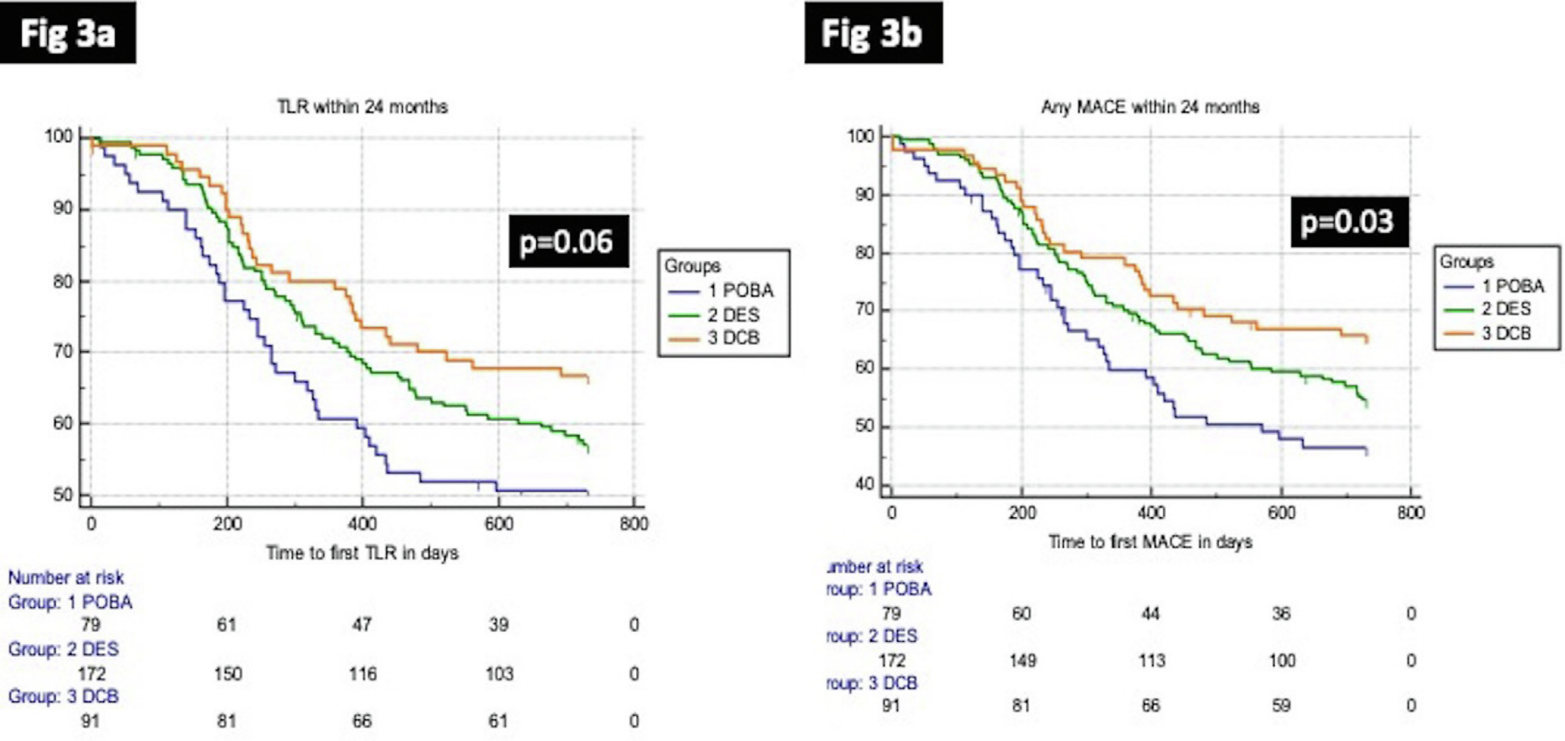
3a and 3b: Kaplan Meier curve of time to TLR and MACE at 24-months between the three treatment arms.

**Fig. 4:**
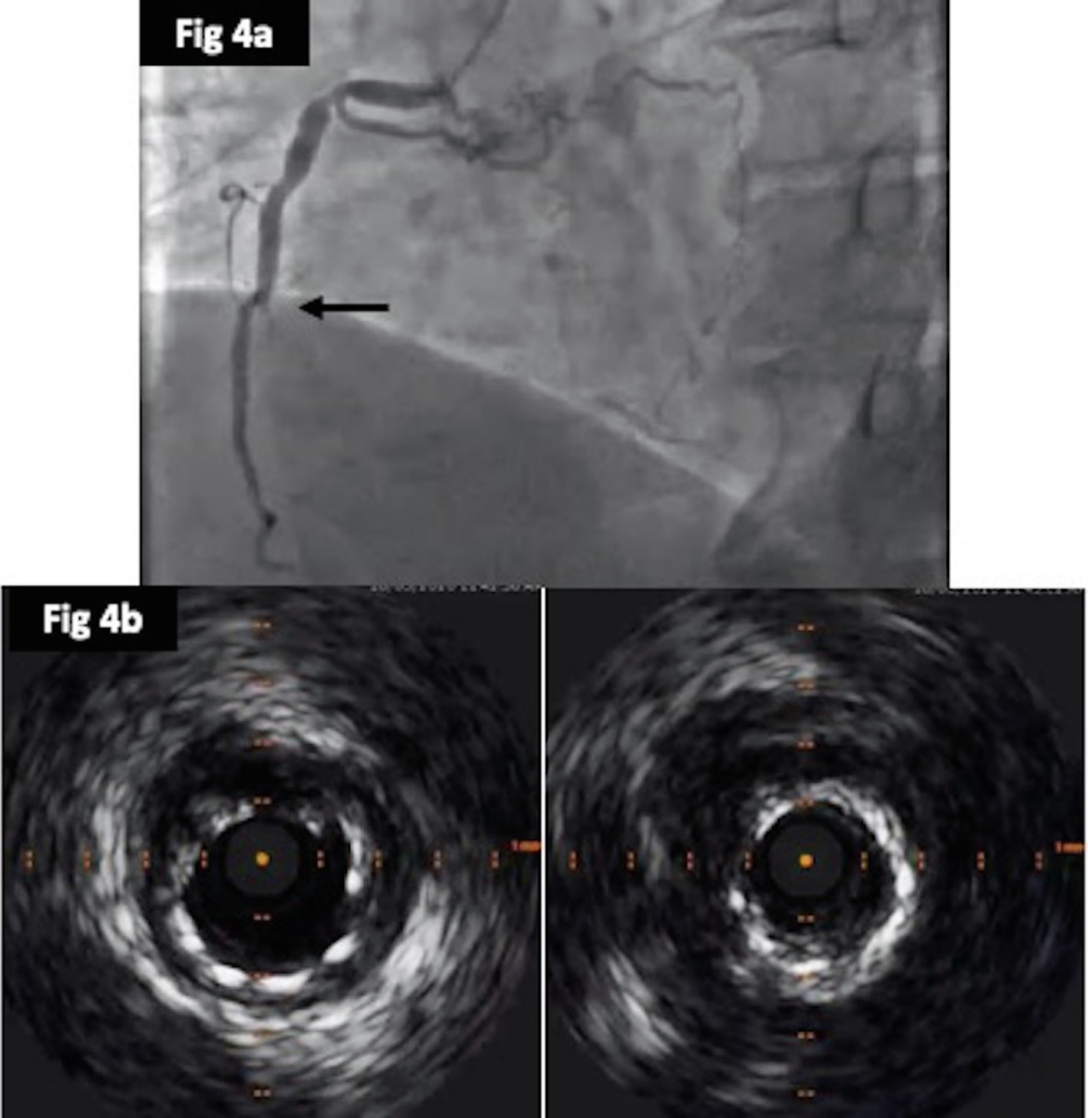
4a: Coronary angiogram showing occluded mid-segment of RCA within the previously stented segment 4b: Intra-vascular ultrasound (IVUS) exhibiting significantly under deployed stent.

**Fig. 5:**
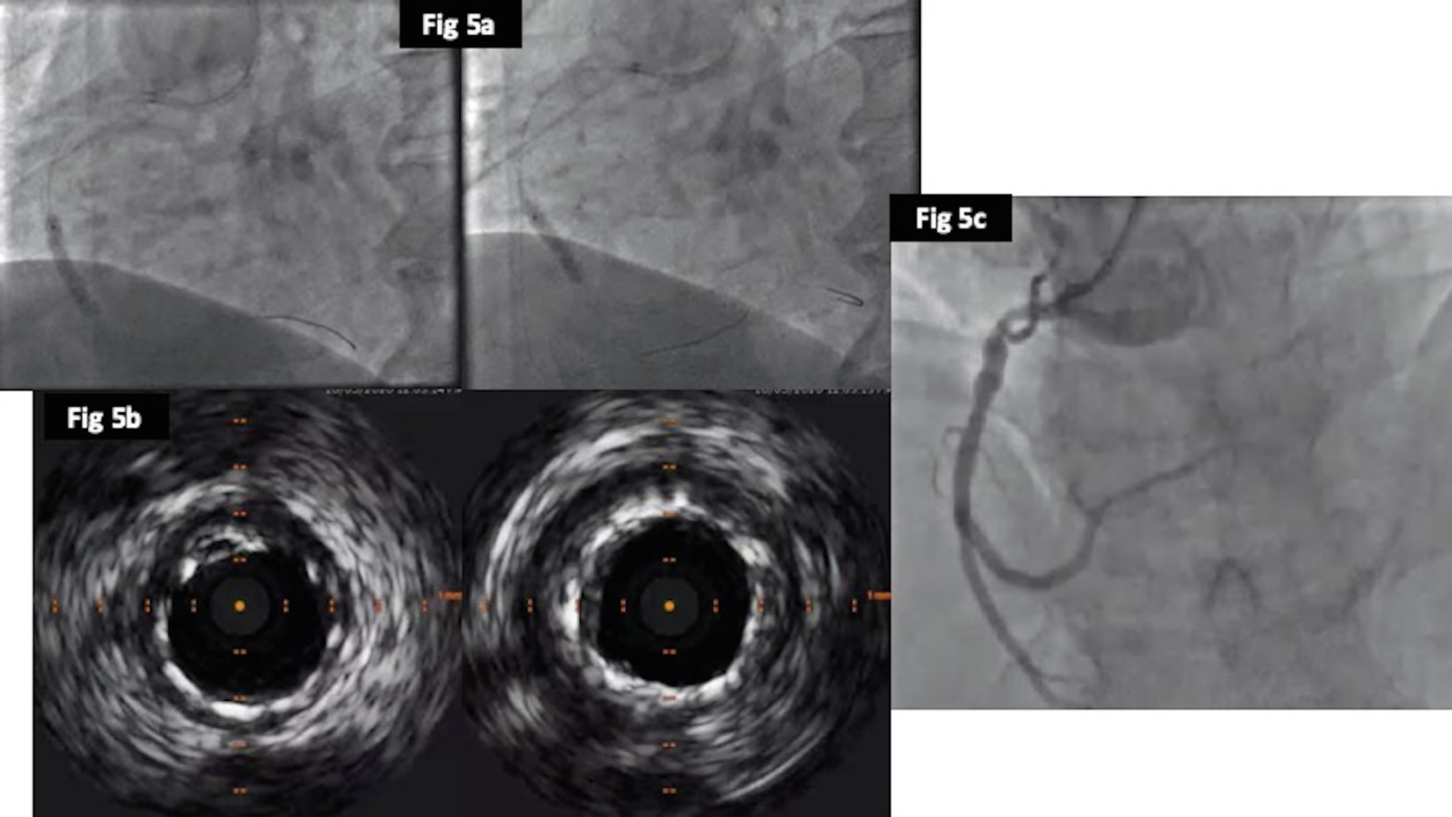
5a: Balloon angioplasty to optimize the stent 5b: IVUS exhibiting significantly better expanded stents 5c: Coronary angiogram showing excellent final result post POBA.

**Fig. 6:**
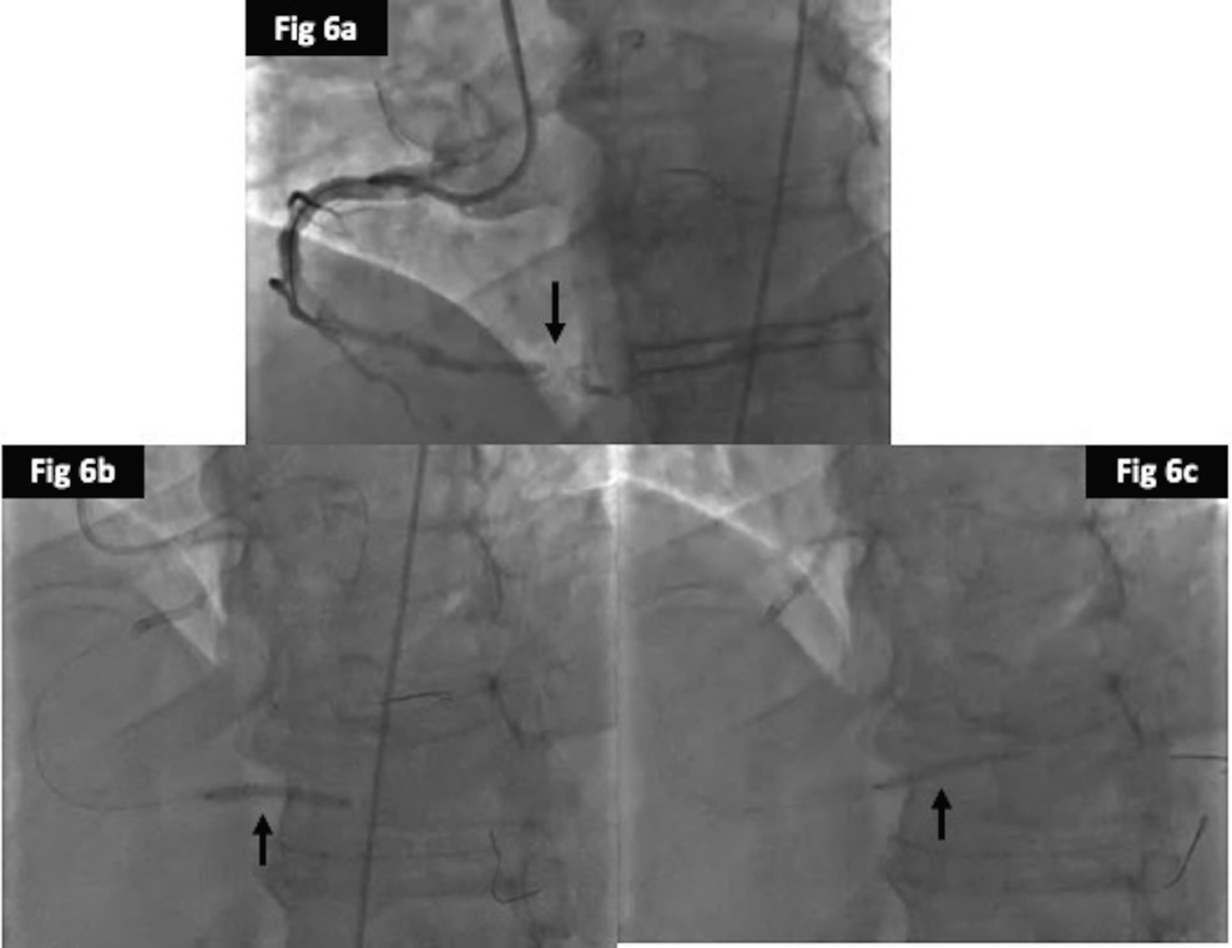
6a: Coronary angiogram showing occluded distal RCA stent at the crux 6b and 6c: Predilatation with non-compliant balloons from distal RCA into both posterior descending and posterior left ventricular branches.

**Fig. 7:**
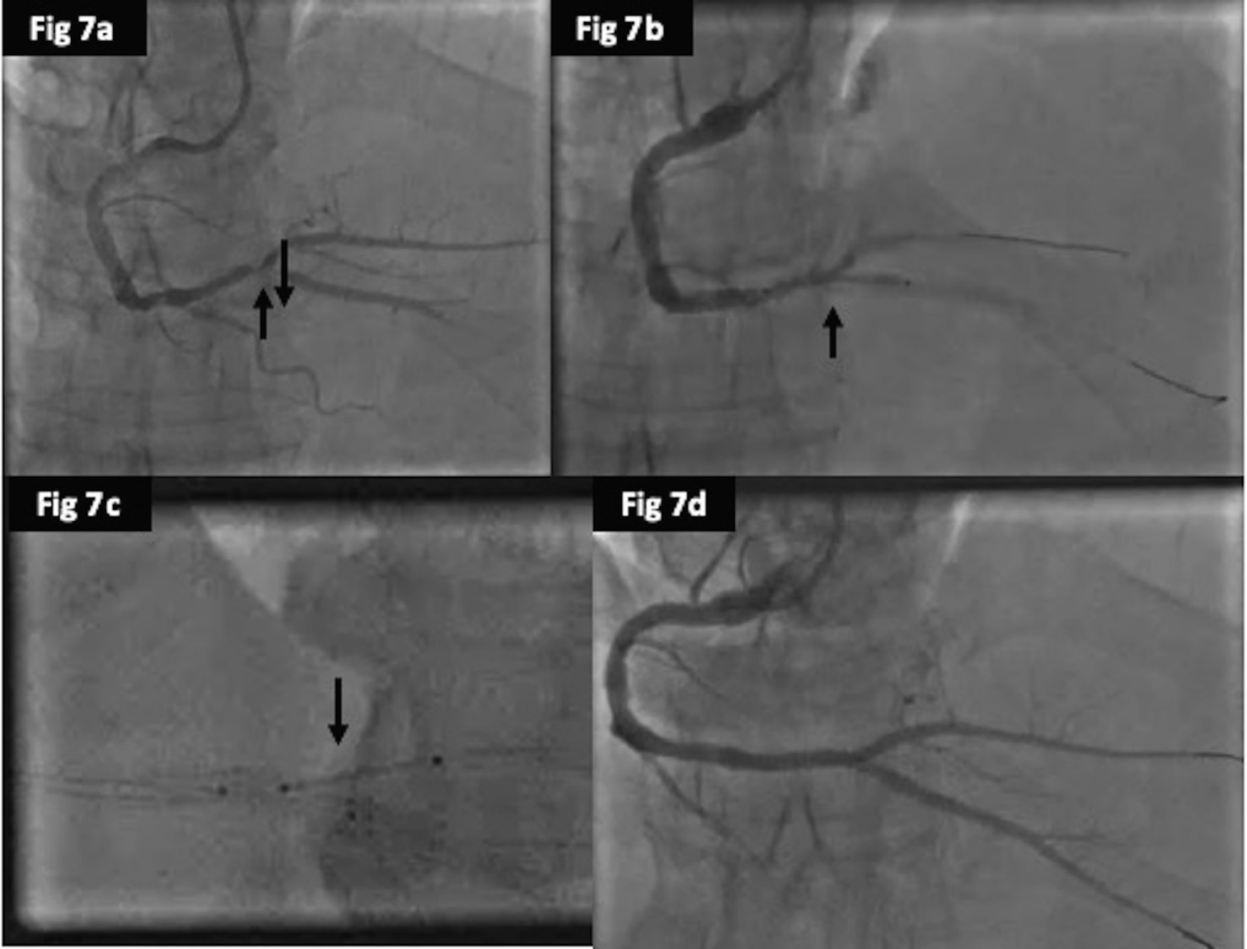
7a: Coronary angiogram showing significant recoil post POBA 7b and 7C: T-and minimal protrusion (TAP) technique to treat the distal RCA bifurcation d: Coronary angiogram showing excellent final result post stenting.

**Fig. 8:**
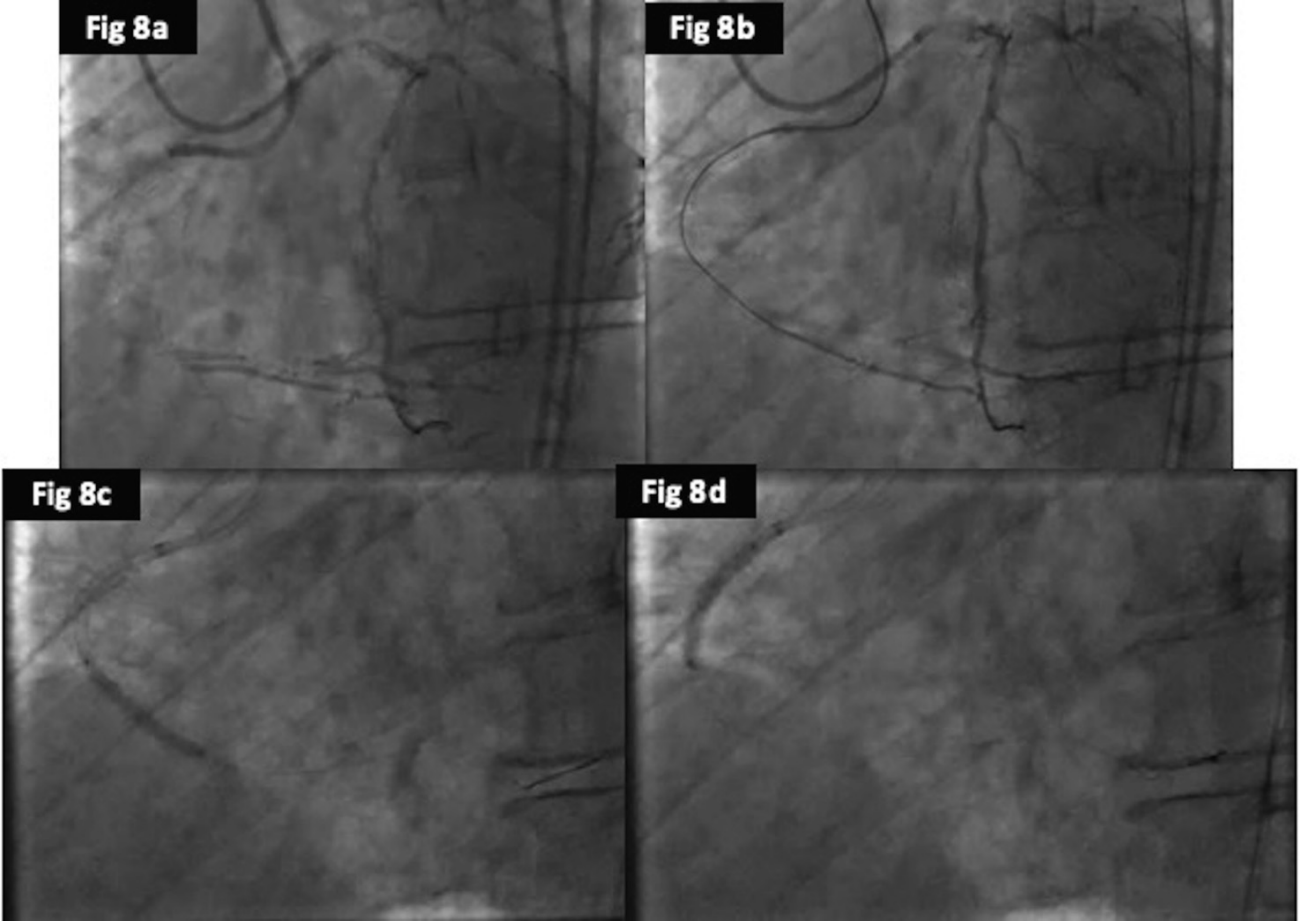
8a: Coronary angiogram showing occluded proximal RCA stent with retrograde collaterals from the left coronary artery. 8b: Successful antegrade wire crossing into the distal RCA confirmed on contra-lateral injection 8c and 8d: Predilatation with non-compliant balloons.

**Fig. 9:**
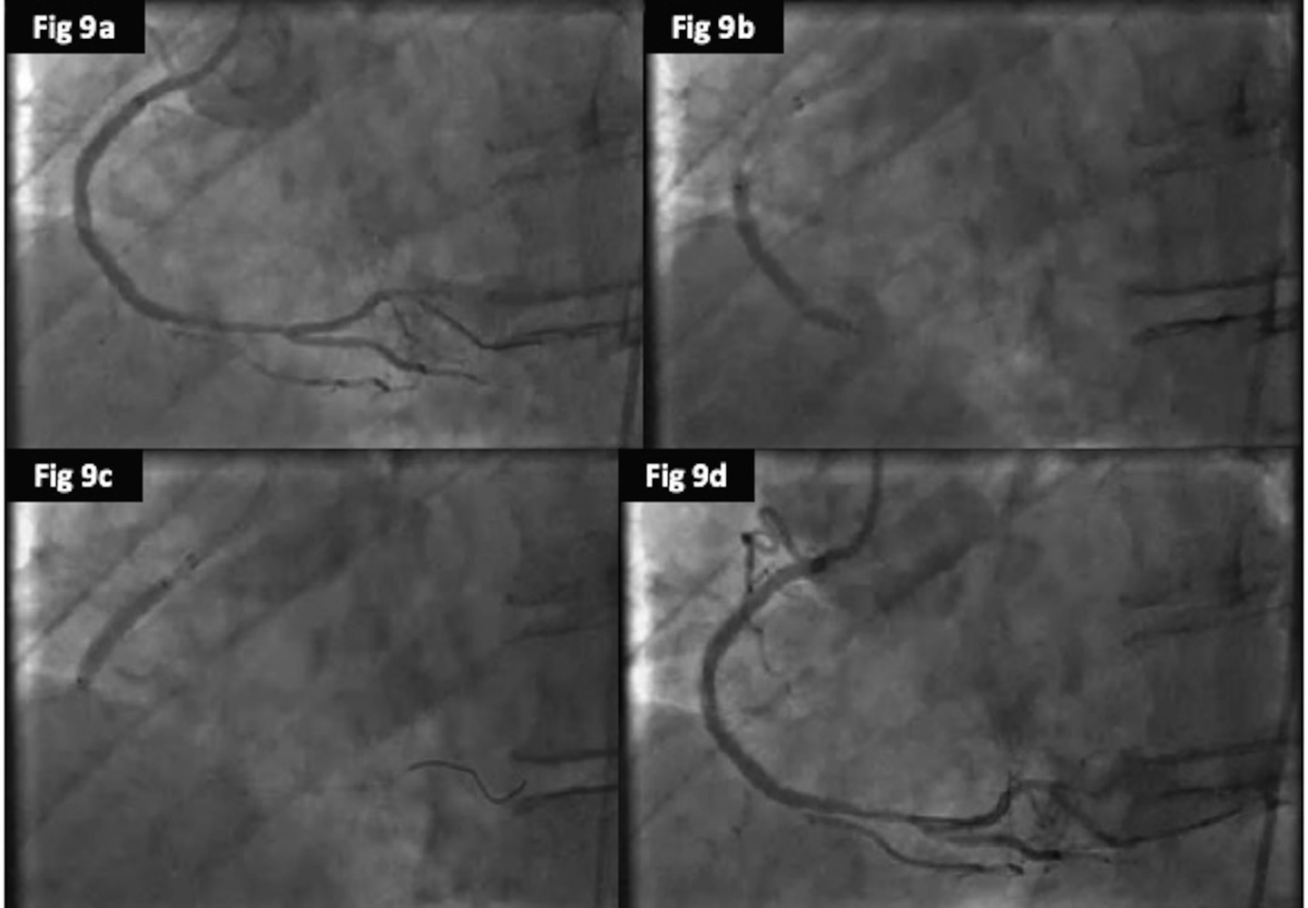
9a: Coronary angiogram post POBA showing no flow limiting dissection or significant re-coil. 9b and 9c: Treatment with 2 overlapping DCBs 9d: Coronary angiogram showing excellent final result.

In regards to clinical outcomes, cardiac deaths were less frequent in the DCB group (1%) as compared to POBA (4%) and DES (3.5%), but was not significant (p = 0.47). There were 3cases of TVMI in DES group, but none in the POBA or DCB, but again it was not significant (p = 0.2). The rates of revascularization (TLR) were better in the DCB (35%) compared to DES (44%) and POBA (53%) and was close to significance (p = 0.06) (Fig. 3a). The overall MACE also favoured the DCB (34%) over DES (45%) and POBA (52%) (p = 0.05). The time to first MACE were significantly shorter for POBA followed by DES and DCB (log rank; p = 0.03) (Fig. 3b). We have provided case examples for each of the treatment modality; POBA (Fig. 4, Fig. 5), DES (Fig. 6, Fig. 7) and DCB (Fig. 8, Fig. 9). The details are provided in the figure legends.

## 7 Discussion

The important findings from this study on long-term clinical outcomes following PCI to intra-stent CTO are:

(1) Acceptable rates of hard endpoints (cardiac death: 5.7% and TVMI: 4%).
(2) High rates of repeat revascularization (TLR: 45% and TVR: 48%) during this long-term follow-up reflecting the complexity of the lesions treated.
(3) DCB angioplasty may have advantages over POBA and DES especially in regards to need for repeat revascularization although selection bias and differences in the procedural characteristics cannot be excluded.

Angioplasty to chronically occluded coronary arteries is escalating owing to consistent improvement in device technology and procedural skills [19]. Although there are several published papers on CTO-PCI, there are relatively limited data on intra-stent CTO [14], [15], [16]. To our knowledge, this is the first large study in the literature of nearly 400 patients that has exclusively focussed on long-term clinical outcomes (with median follow-up of 4years) on occlusive ISR. The complexity of the this highly specialised group is reflected in their demographics with over half of all subjects having diabetes, and approximately one third having CKD and background left ventricular dysfunction. Like most intra-stent CTO-PCI, the antegrade approach predominated, with a very small proportion of patients needing a retrograde approach. Most of the pre-dilatations were carried out with non-compliant balloons with a small proportion of cases needing adjuvant devices such as scoring and cutting balloons. The lesion complexity was reflected in the mean lengths of the DCB and stents employed (48 mm and 37 mm, respectively). We intentionally restricted patient inclusion to 2017 in order to garner long-term follow-up for these patients. During a median follow-up of 4years, the hard endpoints were in an acceptable range (cardiac death 6% and TVMI 4%). However, the TLR and TVR rates were high which drove the MACE rates to 46%, but given the complexity of lesion and patient subsets coupled with the long-term follow-up, these numbers are not surprising. In addition, the angiographic follow-up in our cohort was high (72%), which could partly explain the higher rates of repeat revascularization as an “oculostenotic reflex” may have played a part in it. Nevertheless, as seen in most studies on ISR, the recurrence rates of restenosis are high, and probably more so than for non-occlusive ISR. In the RIBS IV trial, TLR rates at 3years in DCB arm was almost 16% [10]. In the ISAR DESIRE 4 study, which compared the efficacy of scoring balloons in ISR, the TLR rates at 1year was 22% [20]. In a study by Azzalini et al of 111 patients with intra-stent CTO, the MACE rates were over 20% during a median follow-up of over 471days, implying with longer follow-up such as ours, MACE rates would be in a similar range [14]. Considering the high rates of TLR, perhaps PCI to intra-stent CTOs should only be reserved for patients with failed medical therapy and/or if there is a large ischaemic burden (especially in presence of LVSD), as putting patients through a complex, high risk and a lengthy procedure when there is high chance of recurrence may not be justifiable.

One of the other unique features of this study is the comparison of clinical outcomes between the different treatment modalities in intra-stent CTO PCI. There are studies in the literature that have compared these treatment arms but generally in two groups (either POBA vs. stent or POBA vs. DCB or stent vs. DCB) [6], [8], [10], [11], [12]. The only study that has compared all the 3 arms in a single study was ISAR DESIRE 3, but this study had excluded occlusive ISR and also had first generation DES (Paclitaxel) in the stented group, which is not used anymore in clinical practice [6]. In our study, there were no differences in the demographic characteristics between the three groups, but there were some differences in the procedural characteristics. The use of cutting balloons were higher in the POBA group, but despite that DES and DCB appeared to produce better clinical outcomes over POBA. All the other studies that have compared POBA to DCB or DES have all shown the superiority over POBA [8], [11]. Despite these results, POBA is still practiced in several centres either due to non-availability of DCB in clinical practice or due to relative unfamiliarity with the use of DCB. Although our study showed no differences in hard endpoints between POBA and the other treatment modalities (DES and DCB), time to first MACE, which was driven by need for repeat revascularization was significantly shorter in POBA over DES and DCB (p = 0.03). Therefore, we conclude that simple balloon angioplasty after negotiating the arduous and lengthy task of wire passage may not provide adequate treatment for these occlusive restenotic lesions. Even the current European Society of Cardiology guidelines recommends use of DCB or DES in ISR irrespective of the type of restenosis. In our study; the use of POBA were similar in both BMS-intrastent CTO (12%) and DES-intrastent CTO (12%).

In regards to DCB vs. DES, although ESC gives class IA for both treatment modalities, the recent studies have favoured DES especially in regards to better revascularization rates [9], [10], [21]. Although the demographic characteristics were similar between the 2 groups, there were some differences in the procedural characteristics. The diameter of DCB were significantly smaller than the DES diameter, but the length of DES was significantly longer than the DCB, which may counteract any confounding effects on clinical outcomes. In addition, the use of scoring balloons to prepare the lesion were significantly higher in the DCB group, but this is a normal practice in DCB to increase the drug uptake especially when treating ISR. Nevertheless, our results do not show differences in the MACE rates between the DCB and DES, although, numerically DCB had a lower event rates than DES. In addition, the implications of selection bias for such treatment cannot be excluded in our study as the reason to choose one modality over other was not available. Since recruitment dates back to 2011, the data obtained from intra-vascular imaging were not available for all patients to compare between the two groups. Information obtained from the intra-vascular imaging did influence operators on the treatment modality they chose. In general, patients who were treated in the early part of the study received only POBA if the intravascular imaging exhibited under-expanded stent. They were treated with DES if there were no mechanical issue with the previously placed stent and the occlusion was related to intimal hyperplasia and/or neo-atherosclerosis. In the later part of the study, DCB was increasingly used owing to the emergence of more robust data of DCB in the treatment of ISR. The operators opted for DCB if there was a good result following pre-dilatation or stent if there was significant recoil or a flow-limiting dissection. The DCB was used in higher proportion in the DES-intrastent CTO as compared to BMS-intrastent CTO group (32% vs. 12%), which may have also influenced the observed differences between DCB and DES. Nevertheless, it may be argued that implanting a second layer of metal especially in occlusive ISR, often within a long segment of occlusion, will be a recipe for further restenosis and occlusion. So, the modality of treatment has to be carefully selected and the use of DCB may appear pragmatically better especially with optimal pre-dilatation over implanting another layer of stent.

## 8 Limitations

This was a retrospective analysis, which needs to be confirmed in a randomized trial. Since occlusive ISR is rare, randomized trials may not be conceivable. Inview of this, the data such as ours provides insights into the clinical outcomes of intra-stent CTOs and hence aid in implementing better practice. In addition, the rationale for choosing treatment modalities by the operators was not available in all cases. The mode of treatment (DCB vs. DES) might have been decided after the results of pre-dilatation. Although we had high rates of intravascular imaging, unfortunately, the detailed data on imaging was not available in all cases to compare between the treatment groups. Nevertheless, the study population reflects real-world data and provides several messages that will impact upon clinical practice. We had a relatively higher rates of angiographic follow-up, which is not reflective of real-world practice, and this may have had some effect on the rates of repeat revascularisations.

## 9 Conclusion

The results from this study on a large number of patients with intrastent CTO PCI with long-term, and almost complete follow-up provides extremely valuable information that will have an impact on clinical practice. Given the higher rates of TLR and MACE rates, perhaps PCI on this complex group of patients should only be undertaken if there are absolute clinical indications (failed medical therapy and/or a large area of ischemia) as a lengthy, high-risk procedure may not be justifiable. In addition, if PCI is undertaken, DCB and DES should be considered over POBA given the higher rates of TLR and MACE. Between DES and DCB, there were no significant differences, although numerically DCB appears to have favourable results over DES.

## Declaration of Competing Interest

The authors report no relationships that could be construed as a conflict of interest
